# Evaluation of the Change in the Prevalence of Overweight and Obesity in Schoolchildren in South-west Turkey from 2005 to 2014

**Published:** 2018-01

**Authors:** Tuğba KOCA, Selim DERECI, Özgür PIRGON, Mustafa AKÇAM

**Affiliations:** 1. Division of Pediatric Gastroenterology, Hepatology and Nutrition, Faculty of Medicine, Suleyman Demirel University, Isparta, Turkey; 2. Division of Pediatric Endocrinology, Faculty of Medicine, Suleyman Demirel University, Isparta, Turkey

**Keywords:** Childhood obesity, Gender, Overweight, Prevalence, Trend, Turkey

## Abstract

**Background:**

To examine the prevalence of overweight and obesity among schoolchildren in our region and to compare the results with previous studies conducted in the same region in 2005 and 2009.

**Methods:**

This study was conducted at seven primary and three high schools in the center of the province of Isparta, Turkey in 2014, randomly selected for two studies of obesity five and nine years previously. Students were weighed and measured, and BMI was calculated. The results were then compared with those from 2005 and 2009.

**Results:**

The study consisted of 7116 students, 3445 (48.4%) females, and with a mean age of 11.7±2.7 yr (range 5.8–18.9 yr). The prevalence of overweight was 13.6% and that of obesity 9.9%. When the data were compared with the 2005 and 2009 studies, a statistically significant increase was determined in the prevalence of overweight (X^2^ = 4.826, *P*=0.0280 and X^2^ =19.012, *P*<0.0001). The prevalence of obesity in the 2005 and 2009 studies was 11.6% and 12.5%, compared to 9.9% this study. The decrease observed in this study was statistically significant (X^2^=8.720, *P*=0.0031 and X^2^=20.708, *P*<0.0001). The total prevalence’s of overweight and obesity combined were 23.8%, 23.5% and 23.5% for 2005, 2009 and 2014, respectively.

**Conclusion:**

The prevalence of combined overweight and obesity was stable over the nine years, but there was significant increase in the prevalence of overweight. Population-based preventive strategies, therefore, need to be maintained and intensified.

## Introduction

Obesity is an energy metabolism disorder, progressing with excessive fat accumulation in the body and leading to serious physical and psychological problems. Over the past three decades, the prevalence of overweight and obesity has increased substantially (1). Globally, approximately 170 million children (aged fewer than 18) are now estimated to be overweight (2). One-third of overweight children and 80% of overweight adolescents will remain overweight when they reach adulthood (3). Now that obesity is unanimously regarded as a significant health problem worldwide, countries and governments have started to develop and implement policies to combat it. The aim was to achieve a healthier population by slowing the spread of obesity. A reduction in the prevalence is anticipated in countries adopting and implementing such policies.

Even if the acceleration in the growth in the prevalence of obesity is decreasing, obesity itself is still increasing. However, very recent research, particularly conducted in the USA, suggests that an obesity plateau has finally been reached and that the prevalence may now start to decrease (4). In our region, previous studies in our clinic in 2005 and 2009 determined the prevalences of overweight and obesity in children of 12.2%/11.6% and 11%/12.5%, respectively. A slight increase in the incidence of obesity can be seen in that four-year period, but the level of overweight children has fallen (5–7).

The aim of the present study was to examine the changes in overweight and obesity prevalence in Turkish school-age children between 2005 and 2014 and to compare the results with those from previous studies conducted in the same region in 2005 and 2009.

## Materials and Methods

### Participants

The study area consisted of the province of Isparta, located in south-west Turkey of a population of 222556 at the time of the study.

Seven primaries and three high schools stratified by type and city region (central or coastal) were randomly sampled from 146 schools in 2005. All 10 schools participated in both the 2005 and 2009 studies. The same schools were contacted again in 2014 and invited to participate in a third survey. Children who were at school on the days when visits took place were enrolled in the study. The final sample consisted of 7116 schoolchildren.

### Measurements

Overall, 7116 children were measured standing upright in bare feet with heels together using a stable stadiometer. All subjects were weighed on a digital scale, sensitive to 100 gr, with weight recorded in kg (SECA 767, Hamburg, Germany). Subjects stood on the scales with both feet, wearing only school uniform. The scales were recalibrated after each measurement.

### Definitions

Body mass index (BMI) was calculated using the formula body weight (kg)/height (m^2^). BMI percentile charts and tables for age and gender published in 2000 by the Center for Disease Control (CDC) were used in the evaluation of BMI. In terms of BMI percentiles, values <5 were evaluated as underweight, 5–84 as normal, 85–94 as overweight and those ≥95 as obese.

The study was conducted between 20 May and 20 Jun 2014, following receipt of approval from the Local Ethics Committee. The study was conducted in accordance with the Declaration of Helsinki. Informed consent was obtained from the parents of all the children participating in the study.

### Statistical analysis

Descriptive statistics were calculated for all variables (means, standard deviation, and percentages) for the entire sample, based on survey years (2005, 2009, and 2014) and gender. Qualitative data were compared using the Pearson Chi-square test. Statistical analysis was performed on SPSS (ver. 15.0 Chicago, IL, USA) software. Statcalc (Epi Info Version 6) software was used to assess the differences in overweight and obesity percentages between the survey years. *P*<0.05 was regarded as statistically significant.

## Results

The study consisted of 7116 students, 3445 (48.4%) females and 3671 (51.6%) males, with a mean age of 11.7±2.7 yr (range, 5.8–18.9 yr) and mean BMI of 19.1 ±4.0 (range, 10.97–43.9) (Table 1). The total overweight prevalence was 13.6%, and that of obesity 9.9%. Significant differences were determined in the prevalence of overweight and obesity over the preceding nine years. The prevalence of overweight for 2005, 2009 and 2014 was 12.2%, 11.1%, and 13.6%, respectively.

**Table 1:** Distribution of children by gender and age group in each survey year

	2005^*^ N (%)	**2009^#^ N (%)**	**2014 N (%)**	***P*, X^2^**
Gender				
Girls	2447 (51.3)	2454 (42.9)	3445 (48.4)	<0.001, 49.938
Boys	2579 (48.7)	3262 (57.1)	3671 (51.6)	
Age group (yr)				
5.8–10.9	1743^φ^ (34.7)	2857 (49.9)	2745 (38.6)	<0.001, 290.549
>11	3283^φφ^ (65.3)	2859 (50.1)	4371 (61.4)	
Age (mean)	11.8±0.0	11.1±3.1	11.7±2.7	0.765

* Ref 4,

# Ref 5

φ Children <10 yr,

φφ >10 yr

**Fig. 1:**
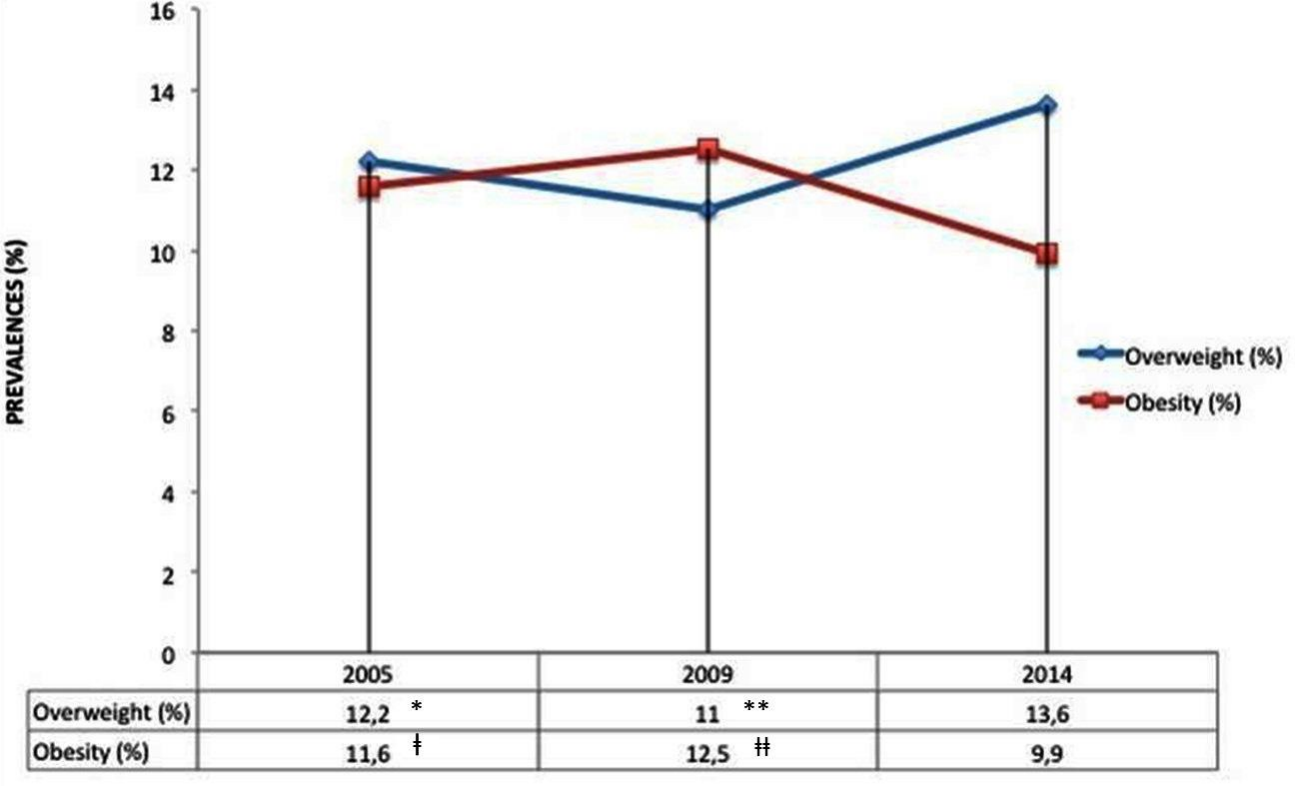
Prevalence of overweight and obesity in each survey year. *P=0.0280 in comparison with this study. **P<0.0001 in comparison with this study. ⱡ*P*=0.0031 in comparison with this study. ⱡⱡ*P*< 0.0001 in comparison with this study.

When the data from this study were compared with the 2005 and 2009 studies, a statistically significant increase was determined in the prevalence of overweight (X^2^ = 4.826, *P*=0.0280 and X^2^ =19.012, *P*<0.0001). The prevalence of obesity in the three studies was 11.6%, 12.5% and 9.9%, the decrease in this study being statistically significant (X^2^=8.720, *P*=0.0031, and X^2^=20.708, *P*<0.0001). The prevalence’s of overweight and obesity combined over the nine years covered by the three studies were 23.8%, 23.5%, and 23.5%, revealing a small decrease in the first five years, but that the rate had remained stable in the last four years (Fig. 1).

In 2005, the prevalence of obesity was 11.1% in children under 10 yr and 11.6% in children over 10 yr. In 2009, the level was 10.2% in children under 11 yr and 14.6% in children over 11. In the present study, the figures were 10.1% and 9.8%, respectively. While the prevalence of obesity in children under 11 yr had not changed in the previous four years, there had been a significant decrease in children aged over 11 (X^2^=39.063, *P*<0.0001).

In males, the prevalence of obesity in 2005, 2009 and 2014 was 14%, 13.4%, and 11.7% respectively. A significant decrease was determined in the prevalence of obesity in the current study compared to the two previous studies (X^2^=4.274, *P*=0.038 and X^2^=11.78, *P*=0.0006). The prevalence of obesity in females was 9%, 11.2%, and 8%, respectively. When the data from the current study were compared with those from 2009, while a statistically significant decrease was determined in both females and males (X^2^=16.826, *P*<0.0001), no significant difference was observed compared to the data from 2005 (X^2^=0.681, *P*>0.05).

## Discussion

The main findings of this study are as follows: 1- the prevalence of overweight increased, 2- the prevalence of obesity decreased, and 3, the combined prevalence of overweight and obesity remained stable between 2005 and 2014 in children aged 6–18 yr.

In a 2007 study from a different province in the same geographical region of Turkey, the prevalence’s of overweight and obesity in primary schoolchildren were similar to the results of the current study at 12.8% and 8.4%, respectively (1). Previous studies of school-age children in Turkey in various provinces at different times have reported levels of overweight in children of 3.7%–17.5% of obesity of 0.9%–10% (8–13).

**Fig. 2:**
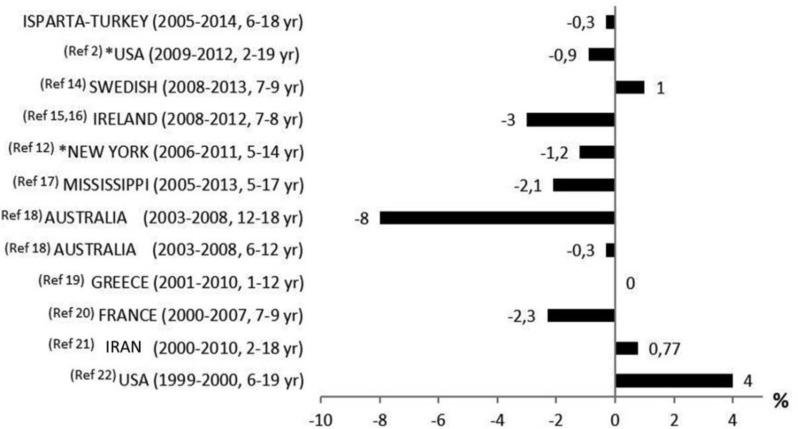
Changes in the prevalence of overweight and obesity in some countries *Changes in the prevalence of obesity

Different classification systems are used, and different age ranges and study periods may be involved to assess childhood overweight and obesity. According to data for 2011–2012 from the American National Health and Nutrition Examination Survey (NHANES), the prevalence of obesity in children aged 2–19 yr was 17.3%, no difference being observed compared to 2009–2010 (18.2%). Although the prevalence of obesity has increased in the last 14 yr, evidence from recent years indicates that this trend is not continuing (4). The prevalence of obesity among children aged 5–14 yr in New York decreased from 21.9% in 2006–2007 to 20.7% in 2010–2011 (14). In England, examination of the prevalence of obesity in 2005–2007 showed that the trend remained fixed in that three-year period (15). The trends of overweight and obesity from some countries are shown in Fig. 2 (4, 14, 16–23).

Prevention strategies for childhood obesity can be implemented through various measures targeting the school environment, physical activity, and diet. Parental awareness and involvement also need to be raised to achieve sustainable changes in support of healthy lifestyles, which can thus make obesity prevention measures more effective (24,25). However, since there was an increase in overweight children, there is no guarantee that the current stability will last or that the prevalence will not increase again in the future.

Childhood obesity is known to exhibit an increase in puberty (26). In our previous study from 2005, no statistically significant difference was observed in the prevalence of obesity among children aged above or below 10 yr. However, a significantly was increased prevalence of obesity in children over 11 (5, 6). In the third and present survey, the prevalence of obesity in children under 11 was higher, although not significantly so. The higher prevalence of obesity in prepubertal children in this study suggests that an adequate and appropriate strategy has still not been developed to prevent obesity in this age group. “The ENERGY-Project study measured BMI across seven European countries and reported that 25.8% of boys and 21.8% of girls were overweight or obese, although the prevalence varied from 14% in girls in Belgium to 44% of boys in Greece” (27). Proportionally more girls than boys are overweight in both developed and developing countries, particularly among adolescents.

In the current study, the prevalence of overweight and obesity were higher in males than in females. Different prevalence’s of obesity in males and females have been reported from various countries. In a study of obesity prevalence and trends in the USA, the prevalence of obesity was significantly higher in males than in females, consistent with the results of the current study (28). In some countries, however, the reverse situation applies, with a higher prevalence being observed in females. Levels of overweight and obesity were higher in females in all studies conducted in Ireland between 2002 and 2012 (29). In Portugal, while studies (30) in 2002 and 2006 reported higher prevalences of overweight and obesity in males, a 2010 study found no significant difference between the genders.

One weakness of this study is that it was conducted in a single region of Turkey. There are several limitations to be considered when interpreting these study results. While data analysis checked for socio-economic status, the level of response within the two ‘school types’ (public and private) may have differed between 2005 and 2014 (this could not be confirmed as these data were not available for 2005).

## Conclusion

Studies on overweight and/or obesity prevalence trends are important. Further research is now needed to investigate whether or not the trend in the stabilization and decline in the prevalence of obesity is continuing. It is also important to monitor the patterns of overweight in order to prevent obesity developing. Finally, in order to raise a healthy new generation, it is essential to acknowledge the key role that schools play in educational measures and to recognize the importance of a well-specified strategy in health promotion programs that include all components of society, health workers, teachers, pupils, families, and institutions.

## Ethical considerations

Ethical issues (Including plagiarism, informed consent, misconduct, data fabrication and/or falsification, double publication and/or submission, redundancy, etc.) have been completely observed by the authors.
